# Deficient Generation of Spike-Specific Long-Lived Plasma Cells in the Bone Marrow After Severe Acute Respiratory Syndrome Coronavirus 2 Infection

**DOI:** 10.1093/infdis/jiad603

**Published:** 2024-02-14

**Authors:** Zahra R Tehrani, Parham Habibzadeh, Robin Flinko, Hegang Chen, Abdolrahim Abbasi, Jean A Yared, Stanca M Ciupe, George K Lewis, Mohammad M Sajadi

**Affiliations:** Institute of Human Virology, University of Maryland School of Medicine, Baltimore, Maryland, USA; Institute of Human Virology, University of Maryland School of Medicine, Baltimore, Maryland, USA; Institute of Human Virology, University of Maryland School of Medicine, Baltimore, Maryland, USA; Department of Epidemiology and Public Health, University of Maryland School of Medicine, Baltimore, Maryland, USA; Institute of Human Virology, University of Maryland School of Medicine, Baltimore, Maryland, USA; University of Maryland Marlene and Stewart Greenebaum Comprehensive Cancer Center, Baltimore, Maryland, USA; Department of Mathematics, Virginia Tech, Blacksburg, Virginia, USA; Institute of Human Virology, University of Maryland School of Medicine, Baltimore, Maryland, USA; Institute of Human Virology, University of Maryland School of Medicine, Baltimore, Maryland, USA; Baltimore VA Medical Center, VA Maryland Health Care System, Baltimore, Maryland, USA

**Keywords:** immunological memory, COVID-19, humoral immunity, plasma cells, SARS-CoV-2

## Abstract

Generation of a stable long-lived plasma cell (LLPC) population is the sine qua non of durable antibody responses after vaccination or infection. We studied 20 individuals with a prior coronavirus disease 2019 infection and characterized the antibody response using bone marrow aspiration and plasma samples. We noted deficient generation of spike-specific LLPCs in the bone marrow after severe acute respiratory syndrome coronavirus 2 infection. Furthermore, while the regression model explained 98% of the observed variance in anti-tetanus immunoglobulin G levels based on LLPC enzyme-linked immunospot assay, we were unable to fit the same model with anti-spike antibodies, again pointing to the lack of LLPC contribution to circulating anti-spike antibodies.

## Teaser

By studying the bone marrow aspiration samples of 20 individuals with prior coronavirus disease 2019, we showed deficient generation of spike-specific long-lived plasma cells in bone marrow as the major contributor to the nondurable humoral immune response to this infection.

## Graphical Abstract

**Graphical Abstract:**
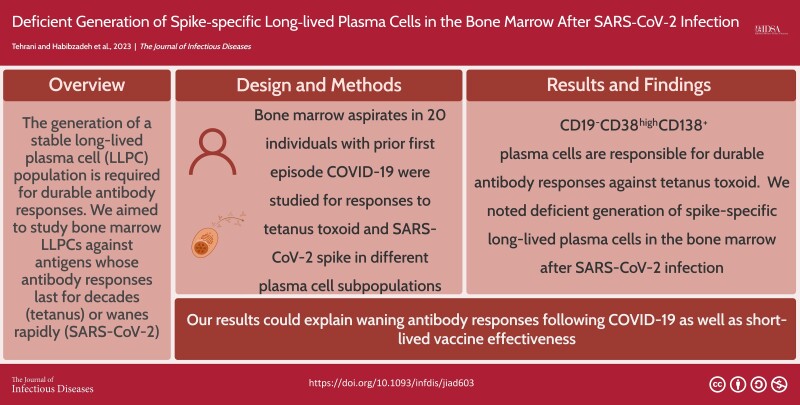

Antibodies are critical components of protection against infectious diseases, including coronavirus disease 2019 (COVID-19). Protective antibodies against viral pathogens such as measles and mumps have been estimated to have half-lives exceeding a human lifetime [1]. By contrast, protective antibodies against other viral pathogens, including influenza and COVID-19, wane rapidly, with half-lives of <1 year [2, 3]. Little is known about why some protective antibody responses persist for decades while others decay quickly. It is known that stable, long-lived plasma cells (LLPCs) in the bone marrow are the sine qua non of durable antibody responses after infection and vaccination. These cells have a distinct morphological and transcriptomic signature and survive in the bone marrow decades after infection or immunization and are the principal component of antibody-based serological memory [4]. Despite emerging findings suggesting the presence of bone marrow spike-specific severe acute respiratory syndrome coronavirus 2 (SARS-CoV-2) infection [5], the lack of information on the phenotypes of these cells and whether they accurately represent LLPCs warrants further investigation.

Understanding the role of LLPCs in serological memory against SARS-CoV-2 and the factors contributing to the generation of these cells provides insight into the durability of the antibody response after natural infection and a powerful tool to predict antibody persistence, eventually mitigating the need for long-term follow-up studies. Herein, we present our findings on the specific plasma cell subpopulations responsible for durable antibody responses (ie, LLPCs) and assess potential mechanisms leading to the deficient generation of spike-specific LLPCs in the bone marrow after SARS-CoV-2 infection.

## METHODS

**Figure 1.**
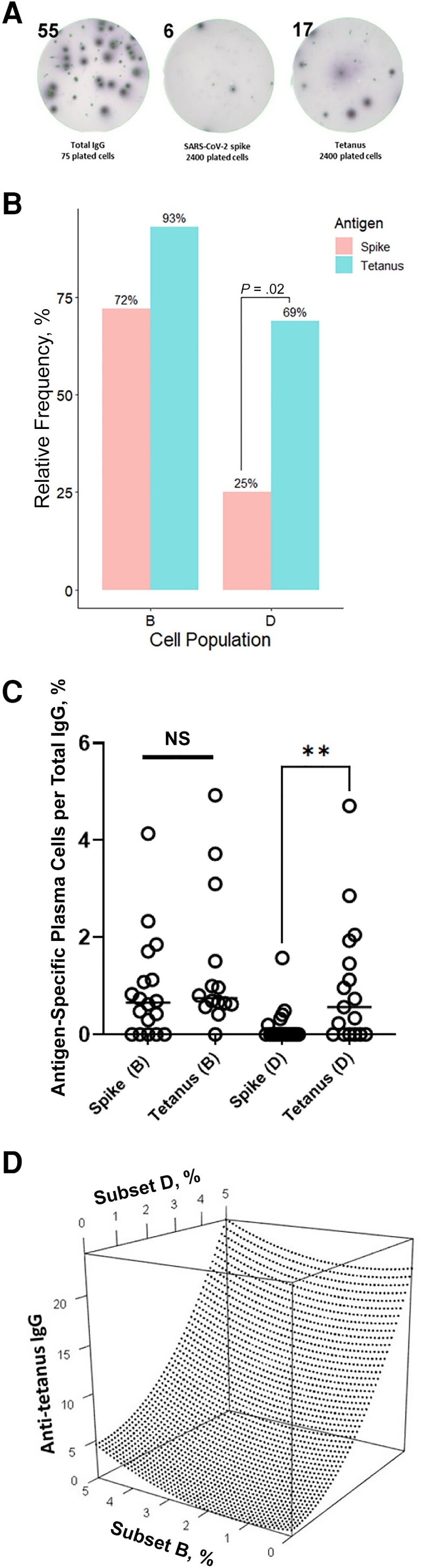
Bone marrow cellular subsets and their contributions to circulating antibody levels. *A,* Bone marrow anti-spike– and anti-tetanus–secreting plasma cells were assessed using enzyme-linked immunospot (ELISPOT) assays. A representative example shows ELISPOT findings for subset B in one of the studied individuals. *B, C,* Flow-sorted plasma cells were plated on severe acute respiratory syndrome coronavirus 2 (SARS-CoV-2) spike trimer or tetanus toxoid plates; qualitative (based on presence or absence of antigen-specific plasma cells) (*B*) and quantitative (based on antigen-specific immunoglobulin [Ig] G–secreting cells: total IgG–secreting cells ratio) (*C*) results are shown (subset B, CD19^+^CD38^hi^CD138^+^; subset D, CD19^−^CD38^hi^CD138^+^). Fisher exact tests were used to compare qualitative results, and Mann-Whitney tests to compare quantitative results. **P = .005. Abbreviation: NS, not significant. *D,* Multivariable regression model plot showing the effect of changes in the percentages of tetanus-specific plasma cells in subsets B and D on circulating IgG levels

This study was approved by the University of Maryland School of Medicine Institutional Review Board. All individuals provided written informed consent before study participation. Volunteers with a history of having COVID-19 once (polymerase chain reaction or antigen positive) and never having received a COVID-19 vaccine had blood collected for plasma and underwent bone marrow aspiration. Bone marrow plasma cell subpopulations with high CD38 expression were isolated based on CD19 and CD138 markers, using flow sorting. Subsequently, enzyme-linked immunospot (ELISPOT) assays for SARS-CoV-2 spike antigen and tetanus antigen were done for the CD19^+^CD38^hi^CD138^+^ (hereafter subset B) and CD19^−^CD38^hi^CD138^+^ (hereafter subset D) cell populations (Figure 1*A* and Supplementary Figure 1). Enzyme-linked immunosorbent assay (ELISA) was used to measure antibody levels for the 2 antigens. Multivariable regression analysis was used to determine the contribution of each of these cellular subsets to the standardized antibody levels against tetanus toxoid (Supplementary Materials). Using mathematical models (see Equation 1 in the Supplementary Materials and Supplementary Figure 4), we identified potential mechanisms explaining the role of LLPCs in antibody production.

## RESULTS

We enrolled 20 healthy individuals who had a history of COVID-19 infection and had never been vaccinated for COVID-19. Of these patients, 30% had a severe infection (see the Supplementary Materials). The sampling was done after a median interval (interquartile range) of 184 (148–250) days following the infection. All patients were infected during the first wave of infection in the United States, from March to November 2020, before arrival of the Alpha, Beta, Delta, and Omicron variants and before the availability of vaccines. Our ELISPOT experiments showed a significantly lower subset D positivity rate against SARS-CoV-2 spike protein than against tetanus antigen (Figure 1*B*), which is known to elicit a long-lived antibody response [4].

Furthermore, the percentage of spike-specific plasma cells per total immunoglobulin (Ig) G–secreting cells was significantly lower than that of tetanus-specific plasma cells among the cells in subset D (Figure 1*C*). Subset D includes the putative cell population responsible for serological memory (ie, LLPCs). In analyses of the 2 donor populations (those with severe vs mild infection), we could not detect anti-spike ELISPOT subset D in the bone marrow aspirates from any patient with past severe infection, while anti-tetanus ELISPOT subset D could be identified in both populations.

While significant correlation was noted between the tetanus-specific plasma subset D percentage and tetanus IgG levels (Pearson *r* = 0.80; *P* < .001), no correlations were observed between the subset B percentage and antibody levels for tetanus or between either of the bone marrow cell subsets and spike IgG titers (Supplementary Figure 2). The regression model could explain 98% of the observed variance in anti-tetanus IgG levels based on the percentage of tetanus-specific ELISPOT assays per total IgG-secreting ELISPOT assays in subsets B and D and demonstrated a significant association between the percentage of tetanus-specific subset D and plasma IgG levels against tetanus toxoid (Figure 1*D*). Importantly, we were unable to fit the subset B and D data with the plasma IgG levels against spike using this model (*R*^2^ = 0.1372).

We showed that when we assume competition between short-lived B cells and LLPCs, with short-lived lineages being recruited at higher rate, <1% of antibody comes from LLPCs, with the majority of the antibody being produced by short-lived B cells 1 year after infection (see Supplementary Figure 4*B,* blue vs red lines). This result is time dependent, with an early maximum LLPC contribution of 7% and no LLPC contribution occurring 1 year after immunization (Supplementary Figure 4*C*, blue vs red lines). This is a representation of a short-lived immunity, as seen in the patients with SARS-CoV-2 infection. By contrast, if we assume no competition for antigen, we have dominance of short-lived B cells early on, owing to their fast priming, but the LLPCs dominate 1 year after the immune response owing to their long life span (Supplementary Figure 4*E,* blue vs red lines). This is a representation of life-long immunity, as seen in the tetanus-vaccinated patients.

## DISCUSSION

We show that unlike tetanus toxoid, which is known to elicit durable antibody responses, SARS-CoV-2 fails to induce a LLPC compartment in the bone marrow. The decrease or defect in spike-specific LLPC generation after SARS-CoV-2 infection could explain waning antibody responses to spike protein after COVID-19 [4]. This also might be the major factor contributing to the waning antibody responses following COVID-19, as well as declining vaccine effectiveness [3].

Furthermore, the absence of subset D cellular population in individuals who had previous severe COVID-19 infection suggests the potential predominance of extrafollicular immune response in these patients, hindering the development of a durable immune memory. This decreased formation of LLPCs in severe infection compared with prior mild disease could be attributable to the predominant activation of the extrafollicular B-cell pathway, which is known to lead to the production of short-lasting antibodies in persons with severe infection [6].

The estimated half-life of antibody responses against tetanus is 11 years. This points toward a relatively stable source of antibody production in the bone marrow (ie, LLPCs) [1]. The significant correlation between the tetanus-specific plasma subset D percentage and the corresponding IgG levels is in line with the notion that plasma cells in subset D are the major contributor to the circulating antibodies long after exposure to tetanus antigen. In contrast, the scanty contribution of these bone marrow cell subsets to COVID-19 antibodies points to the seeming origination of these antibodies as short-lived plasma cells outside the bone marrow. In addition, our regression modeling demonstrating the significant contribution of subset D to the circulating tetanus IgG level as an LLPC subpopulation corroborates the findings of previous studies highlighting the importance of this cell population as a long-term source of antibody production. However, we were unable to fit anti–SARS-CoV-2 spike IgG levels using this model based on the corresponding spike-specific subset B and subset D plasma cell percentages. This again points to the lack of LLPC contribution to the circulating anti-spike antibody levels, and suggests major contributions from other cell populations or compartments that were not measured (eg, lymph nodes).

Finally, our mathematical modeling proposes a testable mechanism of immune regulation, where competition for antigen between LLPCs and faster-expanding short-lived B cells (possibly owing to preexisting immunity to other coronaviruses) may inhibit LLPC expansion, resulting in a skewed phenotypical composition of the B-cell population toward short-lived protection. This could explain the deficient generation of LLPCs in COVID-19.

Despite the vast differences between vaccination for a bacterial pathogen and natural infection with a respiratory virus, in both cases, there is a germinal center reaction in response to the antigen that could ultimately lead to the establishment of a stable plasma cell subpopulation in the bone marrow (ie, LLPCs). Our study was limited by the small sample size, the lack of longitudinal sampling, and the number of antigens studied. Furthermore, other cell populations not studied here (eg, memory B cells) could also contribute to humoral immunity following infection. Further studies are needed to elucidate potential causes for the decreased formation of LLPCs; describe the evolution of this cell population in the bone marrow at different time points before and after infection, leading to the lack of a durable humoral immune response in COVID-19; and explore possible interventions to enhance LLPC formation following SARS-CoV-2 vaccination as well other infectious diseases. In addition, the identification of blood markers associated with the presence of subset D in the bone marrow, enabling noninvasive assessment of a durable humoral immune response, is of great importance.

## Supplementary Data

Supplementary materials are available at *The Journal of Infectious Diseases* online (http://jid.oxfordjournals.org/). Supplementary materials consist of data provided by the author that are published to benefit the reader. The posted materials are not copyedited. The contents of all supplementary data are the sole responsibility of the authors. Questions or messages regarding errors should be addressed to the author.

## Supplementary Material

jiad603_Supplementary_Data

## Notes

***Acknowledgments.*** We thank Xiaoxuan Fan, PhD, University of Maryland Baltimore Flow Cytometry Core, for help with cell sorting, and we also thank the staff at the University of Maryland General Clinical Research Center. We acknowledge the support of the University of Maryland Baltimore, Institute for Clinical & Translational Research (ICTR)/ Clinical and Translational Science Award (CTSA).

***Financial support*.** This work was supported by the National Institutes of Health (grants 2R56AI110259-06, R01AI147870, 5P01AI124912–04, 5TL1TR003100-05); the United States Department of Veterans Affairs (Biomedical Laboratory Research and Development Service Merit Award I01 BX005469-01); the Robert C. Gallo, MD, Distinguished Professorship in Translational Medicine; the University of Maryland, Baltimore, Institute for Clinical & Translational Research; and the National Center for Advancing Translational Sciences Clinical Translational Science Award (grant 1UL1TR003098).
