# Supplementary material for: Deficient Generation of Spike-Specific Long-Lived Plasma Cells in the Bone Marrow After Severe Acute Respiratory Syndrome Coronavirus 2 Infection
Source: J Infect Dis. 2024 Feb 14;230(1):e30–3. doi: 10.1093/infdis/jiad603 (PMC11272043; doi:10.1093/infdis/jiad603)
Supplement: jiad603_Supplementary_Data [file jiad603_supplementary_data.docx]

**Deficient Generation of Spike-specific Long-lived Plasma Cells in the Bone Marrow After SARS-CoV-2 Infection**

Supplemental Material

Research Participants ……………………………………………………………………………………... 2

Cell Processing…………………………………………………………………………………………...... 4

ELISPOT…….…………………………………………………………………………………………...... 6

ELISA……….…………………………………………………………………………………………...... 6

Quantification and Statistical Analysis…….…………….…….………………………………………...... 7

Mathematical Modeling……………..…….…………….…….………………………………………....... 9

References….…………………………………………….….………………………………………….... 13

**Research Participants**

20 Healthy individuals (12 male, 8 female, age range 18-67 years, Median 27 years, IQR 26-46 years) with prior history of COVID-19 infection, which was defined as having a positive SARS-CoV-2 PCR or antigen test, were recruited. Among these patients, 6 had a history of prior severe infection (*i.e.,* those with SpO2 <94% on room air, Pa_O2_/Fi_O2_ < 300 mmHg, respiratory rate > 30 breaths/min, or lung infiltrates > 50%) and 14 had past mild infection (*i.e.,* those with signs and symptoms of COVID-19 such as cough, fever, headache, etc. who do not have shortness of breath or lung infiltrates on imaging) based on NIH definitions (1). EDTA tubes were used to collect the blood samples and the PBMCs were subsequently enriched using density gradient centrifugation. Bone marrow aspiration from the iliac crest was done and approximately 20 ml of bone marrow aspirate was collected in EDTA tubes. The bone marrow sampling was done after a median interval of 184 days (IQR 148-250 days) after infection.

| **Patient ID** | **Age** | **Gender** | **COVID-19 High Risk Factors*** | **Race** | **Disease Severity** | **Time after Tetanus Vaccination (years)** | **Titer Tetanus (IU/mL)** | **Time after COVID-19 (months)** | **Spike EC50** | **Spike-specific subset D ELISPOT positivity** | **Time between COVID-19 and bone marrow aspiration (months)** |
| --- | --- | --- | --- | --- | --- | --- | --- | --- | --- | --- | --- |
| ID533 | 26 | Female | Depression | White | Mild | 5.0 | 2.3 | 5.0 | 163 | - | 5.0 |
| IDHC031 | 26 | Female | None | White | Mild | 5.5 | 1.6 | 6.5 | 876 | - | 6.5 |
| ID518 | 58 | Male | Age > 50 | White | Mild | 0.8 | 4.5 | 8.6 | 421 | + | 8.6 |
| ID534 | 27 | Male | None | White | Mild | 1.5 | 3.6 | 5.1 | 301 | - | 5.1 |
| ID544 | 18 | Male | None | White | Mild | 8.0 | 1.1 | 1.8 | 498 | - | 1.8 |
| ID542 | 27 | Male | Depression | White | Mild | 8.7 | 1.0 | 4.9 | 197 | + | 4.9 |
| ID539 | 26 | Male | None | White/Asian | Mild | 3.4 | 0.6 | 3.6 | 600 | - | 3.6 |
| ID536 | 26 | Female | None | White | Mild | NA | 1.3 | 6.7 | 585 | - | 6.7 |
| ID535 | 25 | Female | None | White | Mild | 4.2 | 2.7 | 6.9 | 553 | - | 6.9 |
| ID538 | 27 | Male | Tobacco (current) | White | Mild | NA | 4.0 | 6.8 | 510 | + | 6.8 |
| ID565 | 28 | Male | Tobacco (former) | White | Mild | NA | 4.1 | 4.9 | 416 | - | 4.9 |
| ID578 | 42 | Male | Obesity, Tobacco (current) | White | Mild | 1.5 | 3.0 | 11.3 | 4606 | + | 11.3 |
| ID577 | 43 | Female | Tobacco (current) | White | Mild | 1.4 | 2.0 | 10.5 | 5835 | - | 10.5 |
| ID579 | 34 | Female | None | White | Mild | 10.1 | 2.1 | 5.4 | 7623 | + | 5.4 |
| ID19 | 67 | Male | DM, Cerebrovascular disease, Tobacco (former), Age > 65 | White | Severe | 2.0 | 4.5 | 9.2 | 1499 | - | 9.2 |
| ID363 | 62 | Male | Age > 50 | White | Severe | 8.4 | 5.2 | 8.6 | 1138 | - | 8.6 |
| ID545 | 47 | Male | DM, Obesity, Tobacco (former) | White | Severe | 5.8 | 19.7 | 7.4 | 709 | - | 7.4 |
| ID546 | 22 | Female | None | White | Severe | 20.4 | 0.6 | 3.3 | 356 | - | 3.3 |
| ID547 | 26 | Female | None | White | Severe | 4.5 | 1.3 | 3.0 | 9406 | - | 3.0 |
| ID549 | 49 | Male | DM, Obesity, Tobacco (former) | White | Severe | NA | 1.6 | 5.8 | 1847 | - | 5.8 |

**Table S1.** Patients demographic, clinical, and laboratory characteristics.

NA= not available

*CDC criteria (Reference 2)

**Cell Processing**

Mononuclear cells were isolated from fresh BM samples using Ficoll density gradient separation. Then the pan B cells were isolated using the EasySep™ Human Pan-B Cell Enrichment Kit (#19554) according to the manufacturer's instructions. After washing with PBS, the cells were incubated with LIVE/DEAD™ Fixable Aqua (ThermoFisher Scientific, #L34965) for 30 minutes at room temperature and protected from light. After washing, the cells were resuspended in FACS buffer (1x Phosphate buffered saline (Ca/Mg2+ free), 1 mM EDTA, 25 mM Hepes, 1% BSA) and stained with the following surface markers: IgD (FITC, Biolegend, #348206), IgM (FITC, Biolegend, # 314506), IgA (FITC, Invitrogen, #H14101), CD3 (FITC, BD Bioscience, #349201), CD14 (FITC, Biolegend, #325604), CD16 (FITC, Biolegend, #302006), CD38 (Alexa Fluor® 647, Biolegend, #303514), CD19 (Brilliant Violet 421™, Biolegend, #302234), CD138 (PerCP-Cy™5.5, Biolegend, #352310) for 30 minutes at room temperature and protected from light. After washing with the FACS buffer, the cells were passed through the 35 µm strainer. Analyzing and sorting the cell populations was performed with a BD FACSAria flow cytometer and sorter (BD Biosciences). Compensation was calculated using single-stained (CD3 marker) cells with the matched fluorophores. Following exclusion of debris, doublets and dead cells, the dump cells (IgD+, IgM+, IgA+, CD3+, CD14+, and CD16+) were gated out and then high CD38 positive cells were sorted based on CD19 and CD138 markers (Supplemental Figure 1). Different cellular subset distributions among CD38+ DUMP negative cells are also reported in Table S2. Cells in subset A and subset C were not used in the subsequent experiments due to their low numbers. The sorted cells were snap-frozen either with BioFlash Drive™ (Fibulas, #C504C4DM) or 10% DMSO, and kept at -80^o^C overnight and then in liquid phase of LN2 tank until to be used for ELISPOT.

| Table S2. Subset Distribution Among Sorted Cells (%) | | | | |
| --- | --- | --- | --- | --- |
| Donor | **A** | **B** | **C** | **D** |
| ID533 | 3 | 57.2 | 0.7 | 39 |
| HC031 | 6.7 | 60 | 1.6 | 31.8 |
| ID518 | 10.3 | 57.8 | 5.2 | 26.7 |
| ID534 | 10.2 | 62.7 | 1.3 | 25.8 |
| ID544 | 18.1 | 69.7 | 0.57 | 11.6 |
| ID542 | 7.2 | 65.7 | 1.7 | 25.4 |
| ID539 | 32.5 | 53.1 | 3.1 | 11.3 |
| ID536 | 7 | 68.2 | 0 | 24.8 |
| ID535 | 42 | 46.9 | 4.9 | 6.2 |
| ID538 | 25.8 | 46.6 | 8.3 | 19.4 |
| ID565 | 56.4 | 30.8 | 4.27 | 8.55 |
| ID578 | 56.1 | 13.9 | 25.3 | 4.7 |
| ID577 | 22.1 | 22.9 | 27.1 | 27.9 |
| ID579 | 22.1 | 51.9 | 5.2 | 20.8 |
| ID019 | 40.7 | 41 | 10.3 | 8 |
| ID363 | 3.1 | 50 | 1.2 | 45.6 |
| ID545 | 20.2 | 66.6 | 4.1 | 9.1 |
| ID546 | 33.5 | 45.8 | 2.8 | 18 |
| ID547 | 35.6 | 52.5 | 5.1 | 6.8 |
| ID549 | 40.6 | 47.1 | 8.24 | 4.12 |


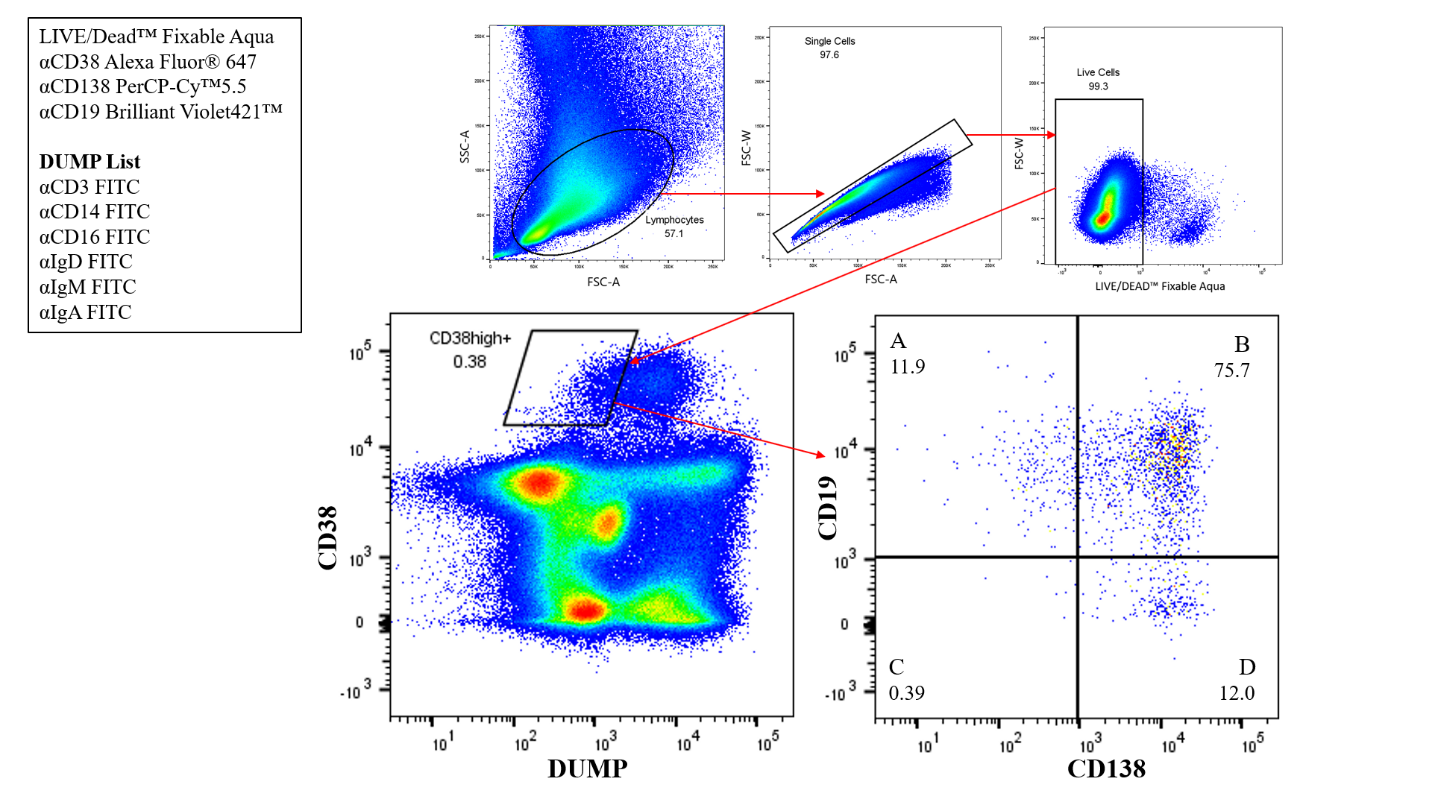


**Fig S1. Isolation of Plasma Cell Subsets**. Different subpopulations of plasma cells were isolated from fresh bone marrow pan B cells. After excluding the duplets, dead, and DUMP cells (CD3+, CD14+, CD16+, sIgD+, sIgM+, sIgA+ cells), the highly expressing CD38 cells were collected into four fractions: (A) CD19^+^/CD138^-^, (B)CD19^+^/CD138^+^, (C) CD19^-^/CD138^-^, (D) CD19^-^/CD138^+^

**ELISPOT**

The sorted plasma cell subsets were added to the 96-well ELISPOT plates (Millipore, #MAHAS4510) coated with tetanus toxoid antigen (2µg/mL, Santa Cruz Biotechnology, #sc-494591), SARS-CoV-2 trimer spike prefusion and trimerization-stabilized ectodomain (2µg/mL, LakePharma, #46328), anti-human kappa/lambda antibodies (2µg/mL of each, SouthernBiotech #2060-01 and #2070-01) and incubated overnight. After washing the wells to remove unbound antibodies, diluted goat antihuman antibody conjugated with alkaline phosphatase (SouthernBiotech #2040-04) was added to each well to detect the bound antibodies. After subsequent washing steps, ELISPOTs were developed by adding BCIP/NBT solution (Mabtech, Cat. #: 3650-10) to the wells. Finally, the spots in each well were counted using ELISPOT reader (S6 Universal M2, Immunospot).

**ELISA**

ELISA for SARS-CoV-2 spike IgG was done using our in-house assay as described previously (3). Briefly, Immulon 2 HB 96-well flat bottom plates (Immuno Chemistry Technologies, Bloomington, MN) were coated with 0.1 μg/well of the trimer spike antigen (LakePharma, Cat. #46328) and incubated overnight at 4^°^C. After blocking all the wells, plasma samples with serial dilutions in Blotto (10% dried milk in TBS and 0.1% NP-40) were added to each well and subsequently the plates were incubated at 37^°^C for one hour. The plates were next washed four times with TBS-T (Tris-buffered saline with 0.1% Tween® 20 detergent) and subsequently incubated with alkaline phosphatase labeled goat anti human IgG (Southern Biotech, Cat. #2040-04) at 37^°^C for one hour. The plates were subsequently washed six times with TBS-T prior to adding BluePhos^®^ Microwell (Seracare, material number 5120-0059) and incubation at 37°C for 15 minutes. The reaction was finally stopped by KPL APstop™ Solution (Seracare, material number 5150-0026) and the optical density was measured at 620 nm (Fisherbrand™ accuSkan, Fisher Scientific). EC50 for each sample was calculated using nonlinear regression. Anti-tetanus toxoid IgG was measured using a commercial Tetanus IgG ELISA Kit (Creative Diagnostics^®^, Cat. # DEIA10378) according to the manufacturer’s instructions.

**Quantification and Statistical Analysis**

In ELISPOT assays, the qualitative analysis was based on the presence of spots in each well for spike or tetanus toxoid antigen. Individual samples in various dilutions were considered replicates. All ELISA experiments were done in duplicates. Pearson's correlation analysis was done to evaluate the association between different antigen-specific plasma cell subsets in the bone marrow and the corresponding plasma antibody concentration (Supplemental Figure 2).


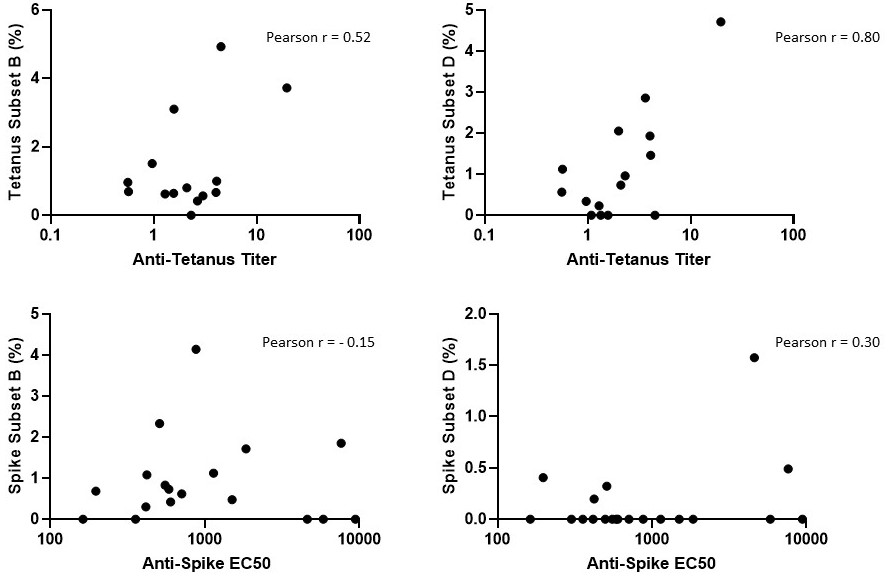


**Fig S2.** Assessing the correlation between different antibody secreting cells percentages and the corresponding plasma antibody levels.

We performed multivariable regression analysis using the least squares method to assess the contribution of different bone marrow cellular subsets in tetanus to the plasma anti-tetanus IgG. Quadratic response surface regression model (4) was used for the modeling and given the small contribution of the cross-product term (parameter estimate of -0.005393), it was excluded yielding the following model:

$${Anti-tetanus IgG Level= \beta0+\beta1 \left( tetanus-specific subset B percentage per total IgG \right)+ \beta2 \left( tetanus-specific subset D percentage per total IgG \right)+ {\beta3 \left( tetanus-specific subset B percentage per total IgG \right)}^{2}+ \beta4 \left( tetanus-specific subset D percentage per total IgG \right)}^{2}$$

Parameter estimates (P values) derived from the Regression analysis (n = 12, R^2^ = 0.982) for β0, β1, β2, β3, and β4 were 1.534 (0.1363), -0.839 (0.3581), 0.065 (0.9393), 0.290 (0.1103), and 0.766 (0.0047), respectively. Shapiro-Wilk test showed that the residuals were normally distributed. Furthermore, anti-tetanus IgG values for various combinations of subset B and D populations percentages were presented in a contour plot (Supplemental Figure 3). Statistical analyses and visualization were done using Prism v.8.4 (GraphPad) and R statistical computing software v.4.2.2. P values of less than 0.05 were considered statistically significant.


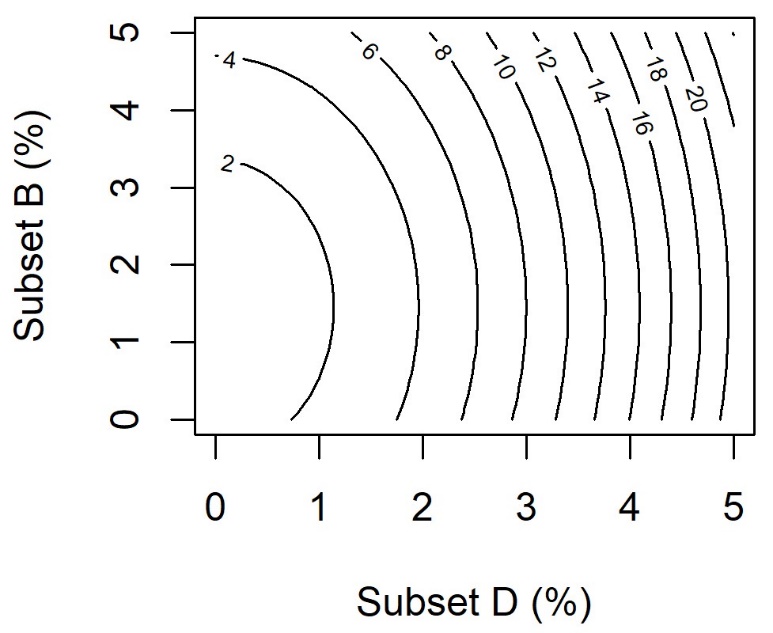


**Fig S3.** Anti-tetanus IgG levels for various combinations of subset B and D population percentages

**Mathematical Modeling**

The immune events and interactions resulting in reduced LLPC production and antibody waning following SARS-CoV-2 infection and/or vaccination and those resulting in long term LLPC persistence following tetanus vaccination are hard to assess experimentally. Mathematical models describing mechanistic interactions of B-cell priming and antibody production in the presence of preexisting and de-nuevo immunity can help in this endeavor. Here, we adapt previous modeling approaches used in influenza studies (5,6) to propose hypotheses explaining the differing LLPC outcomes presented in this study. Briefly, as in mathematical models of influenza (5,6) we hypothesized that challenge with novel viral strains may lead to the priming of epitope-specific short-lived B-cells rather than broad (or strain)-specific long-lived plasma cells (LLPCs) and that competition between B-cell lineages shapes the immunity landscape. To address this we developed a mathematical model of B-cell priming that assumes the interaction between free antigen, $H_{f}$; short-lived B-cells specific to a variable antigen epitope, $B_{1}$; LLPC specific to conserved antigen epitope, $B_{2}$; antibody produced by each B-cell class, $A_{1}$ and $A_{2}$; and immune complexes formed by free antigen binding by either one antibody or both, $H_{b1}$, $H_{b2},$ and $H_{b}$ (Supplemental Figure 4A; Supplemental Table S3). We assume that antibodies $A_{1}$ and $A_{2}$ bind to free antigen $H_{f}$ and bound antigen $H_{b1}$ and $H_{b2}$at the same rate $k={10}^{-3}$ per antibody per day and decay at rate $d=0.5$ per day. Both free antigens and immune complexes prime strain-specific B-cells. We model this using a density dependent term with per capita priming rate, $s_{i}$ (i=1,2); half-maximal activation $\phi=300$ per ml; and reduced priming rate by the non-specific antigen, $\delta=0.5.$ We assume higher priming rate for the short-lived B-cells compared to LLPC, $s_{1}>s_{2}$ and, without loss of generality, we let $s_{1}=10s_{2}.$ Short lived B-cells have a life-span of 2.5 months ${(d}_{1}$=0.013 per day) and the LLPC have a life-span of 2 years ($d_{2}$=0.0013 per day). The two types of B-cell lineages compete with each other for immunodominance (due to either the effects of precursor frequencies, antigen affinity and avidity, and/or the strength of T cell help (7)) at rate $\mu$. Both B-cell types produce the same amount of antibody, $a=1$ per day. Lastly antibody has a life-span of twenty days$, d_{A}=0.05$ per day. Put together these assumptions result in the following system:

(eq. 1)

Initially, there is small (equal among classes) initial B-cell concentration and antibody titer $B_{1}\left( 0 \right)=B_{2}\left( 0 \right)=A_{1}\left( 0 \right)=A_{2}\left( 0 \right)=1$ per ml, large free antigen concentration $H_{f}=30\times{10}^{5}$ per ml and no bound antigen $H_{b1}=H_{d2}=H_{b}=0$ per ml. Parameters {$k,d,d_{1},d_{2},\phi,\delta,a,d_{A}$} are assumed known (Table S3 and above text) and parameters {$s_{1},s_{2},\mu$} are assumed unknown, with the caveat that $s_{1}=10s_{2}$. We estimate the unknown parameters {$s_{2},\mu$} by fitting model (eq. 1) against antibody titer data from an antibody negative, COVID-19 immunized patient (patient 26 data, published in (8)). We fitted the total antibody population given by $A_{1}\left( t \right)+A_{2}\left( t \right)$ in model (eq. 1) to the antibody titer data using the ‘fminsearch’ algorithm in MATLAB and assumed $s_{1}/s_{2}=10$. The resulting parameter estimates are given in Table S3 and the antibody and B cell dynamics are shown in Supplemental Figure 4 (panels **B-C**). We found that, while the total antibody titer $A_{1}\left( t \right)+A_{2}\left( t \right)$ matches the patient data (see Supplemental Figure 4**,** panel **B** solid black line versus black dots), less than 1% of antibody comes from LLPC (class $B_{2}$), with the rest of the antibody being produced by short lived B-cells (class $B_{1}$) (see Supplemental Figure 4 panel **B**, blue versus red line). This result is time-dependent, with early maximum 7% LLPC contribution and no LLPC contribution occurring one year after immunization, for the parameters considered in the model (Supplemental Figure 4 panel **C,** blue versus red line). This is a representation of a short-lived immunity, as seen in the SARS-CoV-2 patients. By contrast, if we assume no competition for antigen ($\mu=0)$, we have dominance of strain specific B-cells early on, due to their fast priming but the LLPC dominate one year after immune response due to their long life-span (Supplemental Figure 4, panel **E,** blue versus red line). This is a representation of a life-long immunity, as seen in the tetanus vaccination patients. Hence, model (eq. 1) proposes a testable mechanism of immune regulation, where competition for antigen between faster expanding of short-lived B-cells (possibly due to preexisting immunity to other coronaviruses) may inhibit LLPC expansion, resulting in a skewed phenotypical composition of the B-cell population towards short-lived protection.

| **Variable** | **Variable description** | **Initial value** |
| --- | --- | --- |
| $H_{f}$ | Free antigen | $3\times{10}^{5}$ per ml |
| $H_{b1}$ | Antigen bound by antibody 1 | 0 per ml |
| $H_{b2}$ | Antigen bound by antibody 2 | 0 per ml |
| $H_{b}$ | Antigen bound by both antibody | 0 per ml |
| $B_{1}$ | Short-lived B-cells | 1 per ml |
| $B_{2}$ | LLPC | 1 per ml |
| $A_{1}$ | Antibody produced by short-lived B-cells | 1 per ml |
| $A_{2}$ | Antibody produced by LLPC | 1 per ml |
| **Parameter** | **Parameter description** | **Value (fixed)** |
| $k$ | Antibody binding rate | ${10}^{-3}$ $d^{-1}$ |
| $d$ | All antigen decay rate | 0.5 $d^{-1}$ |
| $d_{1}$ | Clearance rate of short-lived B-cells | 0.013 $d^{-1}$ |
| $d_{2}$ | Clearance rate of LLPC | 0.0013 $d^{-1}$ |
| $\delta$ | Relative B-cell activation by non-specific antigen | 0.5 |
| φ | Antigen where B-cell priming is half-maximal | 100 |
| $a$ | Antibody production rate | 1 $d^{-1}$ |
| $d_{A}$ | Antibody decay rate | 0.05 $d^{-1}$ |
| $s_{1}$ | Priming of short-lived B-cells | $10$ $\times s_{2}d^{-1}$ |
| **Parameter** | **Parameter description** | **Value (estimated)** |
| $s_{2}$ | Priming of LLPC | 43.1 $d^{-1}$ |
| $\mu$ | Competition rate between B-cells | $8\times{10}^{-4} d^{-1}$ |

**Table S3.** Description and values for variables and parameters used in the mathematical model eq (1).


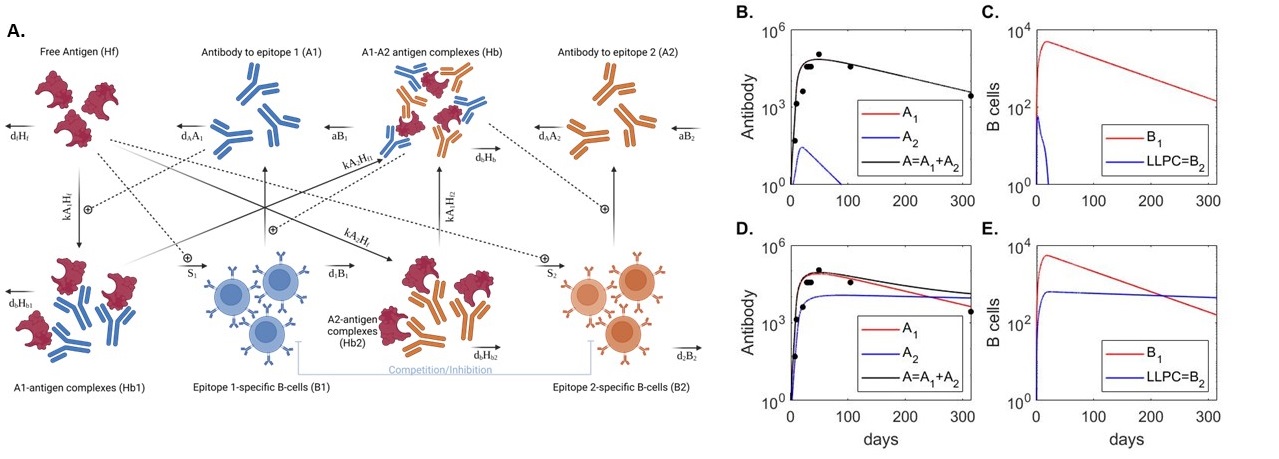


**Fig S4. A.** Diagram of the model (eq. 1). **B-E.** Dynamics of the mathematical model (eq. 1). Panels **B-C** represent B-cell populations and antibody concentrations over time when there is competition for antigen between B-cell lineages (μ=8$\times{10}^{-4})$and panels **D-E** represent B-cell populations and antibody concentrations over time when there is no competition for antigen between B-cell lineages (μ=0). The black dots are data from an antibody negative, immunized patient in (8). The black lines correspond to total antibody titers ($A_{1}+A_{2}$), blue curves correspond to LLPC and antibody titers from LLPC priming ($B_{2}$and $A_{2})$, and the red curves correspond to short-lived B-cells and antibody titers from short-lived B-cell priming ($B_{1}$and $A_{1})$. Note that in panel **B** the red and black curves overlap. For the time period considered, the LLPC account for maximum of 7% of the total anti-spike antibody response under antigenic competition and 100% of the total anti-spike in the absence of competition.

**References**

1. COVID-19 Treatment Guidelines. <https://www.covid19treatmentguidelines.nih.gov/overview/clinical-spectrum/>. Accessed 1/3/2023, 2023.

2. People with Certain Medical Conditions. <https://www.cdc.gov/coronavirus/2019-ncov/need-extra-precautions/people-with-medical-conditions.html/>. Accessed 2/14/2023, 2023.

3. Rikhtegaran Tehrani Z, Saadat S, Saleh E, Ouyang X, Constantine N, DeVico AL, et al. Performance of nucleocapsid and spike-based SARS-CoV-2 serologic assays. *PLoS One.* 2020;15(11):e0237828.

4. Myers RH, Montgomery DC, and Anderson-Cook CM. *Response Surface Methodology: Process and Product Optimization Using Designed Experiments.* Wiley; 2016.

5. Zarnitsyna VI, Lavine J, Ellebedy A, Ahmed R, Antia R (2016) Multi-epitope Models Explain How Pre-existing Antibodies Affect the Generation of Broadly Protective Responses to Influenza. *PLoS Pathog*. 2016; 12(6): e1005692.

6. Linderman, S. L., Ellebedy, A. H., Davis, C., Eberhardt, C. S., Antia, R., Ahmed, R., et al. Influenza immunization in the context of preexisting immunity. Cold Spring Harbor perspectives in medicine. 2021; 11(11), a040964.

7. Abbott RK, Crotty S. Factors in B cell competition and immunodominance. Immunol Rev 2020; 296:120-31.

8. Sajadi, M. M., Myers, A., Logue, J., Saadat, S., Shokatpour, N., Quinn, J., et al. Mucosal and Systemic Responses to Severe Acute Respiratory Syndrome Coronavirus 2 Vaccination Determined by Severity of Primary Infection. Msphere. 2022;, 7(6), e00279-22.
